# Efficacy and Tolerability of a Food Supplement Based on *Zea mays* L., *Gymnema sylvestre* (Retz.) R.br.ex Sm, Zinc and Chromium for the Maintenance of Normal Carbohydrate Metabolism: A Monocentric, Randomized, Double-Blind, Placebo-Controlled Clinical Trial

**DOI:** 10.3390/nu16152459

**Published:** 2024-07-29

**Authors:** Daniele Giuseppe Buccato, Hammad Ullah, Lorenza Francesca De Lellis, Maria Vittoria Morone, Danaé S. Larsen, Alessandro Di Minno, Marcello Cordara, Roberto Piccinocchi, Alessandra Baldi, Agostino Greco, Salvatore Santonastaso, Roberto Sacchi, Maria Daglia

**Affiliations:** 1 Department of Pharmacy, University of Napoli Federico II, Via D. Montesano 49, 80131 Naples, Italy; d.buccato@studenti.unina.it (D.G.B.); hammadrph@gmail.com (H.U.); lo.delellis2@libero.it (L.F.D.L.); alessandra.baldi.alimenti@gmail.com (A.B.); maria.daglia@unina.it (M.D.); 2 Department of Experimental Medicine, Section of Microbiology and Clinical Microbiology, University of Campania “L. Vanvitelli”, 80138 Naples, Italy; mariavittoria.morone@unicampania.it; 3 School of Chemical Sciences, The University of Auckland, Auckland 1010, New Zealand; d.larsen@auckland.ac.nz; 4 CEINGE-Biotecnologie Avanzate, Via Gaetano Salvatore 486, 80145 Naples, Italy; 5 School of Medicine, University of Milano-Bicocca, 20126 Milan, Italy; m.cordara@campus.unimib.it; 6 Level 1 Medical Director Anaesthesia and Resuscitation A. U. O. Luigi Vanvitelli, Via Santa Maria di Costantinopoli, 80138 Naples, Italy; roberto.piccinocchi@policliniconapoli.it; 7 A.S.L. Caserta, Via P. Harris, 81100 Caserta, Italy; agostino.greco@alice.it (A.G.); salvatore.santonastaso@tin.it (S.S.); 8 Applied Statistic Unit, Department of Earth and Environmental Sciences, University of Pavia, Viale Taramelli 24, 27100 Pavia, Italy; roberto.sacchi@unipv.it; 9 International Research Center for Food Nutrition and Safety, Jiangsu University, Zhenjiang 212013, China

**Keywords:** food supplement, vegetable extracts, essential microelements, glucose metabolism

## Abstract

A study on 81 individuals (18–75 years old) with mildly impaired fasting blood glucose (FBG) concentrations (98–125 mg/dL) was undertaken to investigate the tolerability of a food supplement (FS) based on *Zea mays* and *Gymnema sylvestre* extracts, zinc, and chromium and its efficacy on glucose and lipid metabolism. The subjects were randomized into three groups (27 in each group) and supplemented with one or two tablet(s)/day of FS (groups 1 and 2, respectively), or two tablets/day of placebo (group 3). Blood sampling was carried out at baseline (t0) and after a 3-month treatment (t1), and biochemical parameters associated with glucose and lipid metabolism and kidney and liver toxicity were evaluated. Compared to the placebo, FBG and glycated haemoglobin (HbA1c) were significantly (*p* < 0.001) reduced in group 1 subjects. In contrast, at the doses of one and two tablet(s)/day, the FS exerted no effect on the other parameters examined. We conclude that in subjects with slightly impaired FBG, ingestion of a FS based on *Z. mays* and *G. sylvestre* extracts, zinc, and chromium over 3 months lowers FBG and modulates glucose homeostasis by improving glucose metabolism. These beneficial effects occur in the absence of biochemical evidence of kidney and liver toxicity.

## 1. Introduction

Metabolic syndrome (MetS) is characterized by chronic low-grade inflammation due to inherited and environmental factors (e.g., hyperlipidemia, high blood pressure, visceral obesity, and abnormal blood glucose levels) [1,2]. MetS can silently progress towards diabetes mellitus type 2 (T2DM), coronary artery disease, heart failure, stroke, and liver steatosis and steatohepatitis [3]. Dysregulation of adipose tissue and an imbalanced secretion of pro- and anti-inflammatory adipokines are critical in MetS and contribute to the clinical complications of this condition [4]. Given the diverse range of risk factors involved and the complications that can develop over time, MetS is classified as a widespread, multifactorial disease with significant variation across different populations [5].

Lifestyle modifications, especially dietary habits, can significantly reduce the prevalence of MetS. Compared to low-fat and highly restrictive diets, the Mediterranean diet has emerged as a paradigm for preventing and treating MetS [6]. Its macro- and micro-nutrient and bioactive compound compositions positively influence MetS markers [7]. On the other hand, by reducing risk factors associated with MetS, such as by increasing physical activity and fitness, dramatic improvement in insulin resistance, lipid disorders, or obesity can be observed [8]. However, when a healthy diet and lifestyle changes do not suffice to control the risk factors associated with MetS, drugs to improve blood pressure, insulin sensitivity, blood glucose, cholesterol and triglyceride levels are needed to prevent severe complications of MetS [9].

Food supplements are a safe and efficient alternative to prevent side effects often associated with the use of drugs to lower risk factors for MetS, and are a viable strategy to increase the adherence to healthy diets and lifestyle changes such as weight loss and physical activity. Bioactive food supplement ingredients used to improve carbohydrate and lipid metabolism, either in single or mixed forms, include plants extracts (e.g., *Morus alba* L., *Mangifera indica* L., *Gymnema sylvestre* (Retz.) R.Br. ex Sm., *Cynara scolymus* L., and *Coffea* species) [10,11,12,13,14] and essential trace elements (e.g., zinc, magnesium, vanadium, chromium, and selenium) [15,16].

Moradyn^®^ (Italian purple corn), rich in flavonoids and hydroxycinnamic acids, has emerged as a potential agent to inhibit the formation of advanced glycation end products (AGEs) [17]. AGEs are critical in the pathophysiology of diabetic complications. Moreover, Moradyn^®^ inhibits the activity of enzymes involved in carbohydrate digestion, blocking starch digestion and reducing glucose absorption [17]. Among the botanicals proposed as potential ingredients for glycemic control, the leaves of *G. sylveste* (Apocynaceae) are very promising [18]. The bioactive compounds present in the plant leaves include saponins, polysaccharides, sterols, terpenoids, flavonoids, peptides, and pectin [19]. *G. sylvestre* extracts (i.e., aqueous, methanol, ethanol, and acetone extracts) have been evaluated for treating obesity and diabetes mellitus in both animal and human studies with beneficial effects on these conditions [20,21,22,23].

Zinc and its carrier proteins are crucial for the synthesis and secretion of insulin, as well as for the signaling pathways regulating insulin activity [24,25]. Accordingly, zinc deficiency is associated with glucose intolerance and insulin resistance [26]. Zinc is also linked to risk factors for diabetes and CVD. Indeed, visceral fat accumulation in obese individuals leads to high cortisol synthesis, which in turn lowers the expression of proteins regulating plasma zinc levels [27]. Zinc depletion is a critical factor in hepatic steatosis, while its depletion is correlated with dysfunctional peroxisome proliferator-activated receptors (PPAR-α) and triggers lipid peroxidation reactions [16].

Chromium is an essential trace mineral involved in glucose and fatty acid metabolism that plays a vital role in insulin secretion. Chromium is also present in the diet and may increase insulin secretion, causing hyperinsulinemia, insulin resistance, and weight gain [28,29].

The present study aimed to test whether a FS containing a combination of ingredients (*Z. mays* and *G. sylvestre* extracts, zinc, and chromium) acting at different levels in the body could help to restore physiological glucose and lipid metabolism. We have also assessed the tolerability of prolonged use of this FS by measuring creatinine (CRE) and transaminases (ALT and AST) levels before and after the food supplement treatment.

## 2. Materials and Methods

### 2.1. Materials

According to the current European legislation, “*food supplements’ means foodstuffs the purpose of which is to supplement the normal diet, and which are concentrated sources of nutrients or other substances with a nutritional or physiological effect, alone or in combination, marketed in dose form*” [30]. The FS studied in the present clinical trial was produced in accordance with this definition by Gricar Chemical S.r.l. and formulated with a blend of key ingredients: *G. sylvestre* hydroethanolic extract, standardized to contain 25% gymnemic acids; Moradyn^®^ hydroethanolic extract derived from purple maize varieties (*Z. mays*), standardized to contain 10% polyphenols; 3% anthocyanins and anthocyanin derivatives (its metabolic profile has been studied in depth by Ferron et al. [17]); and zinc oxide and chromium picolinate to enrich its composition. Microcrystalline cellulose and calcium phosphate dibasic serve as bulking agents. To prevent clumping, the formula also includes silicon dioxide and magnesium salts of fatty acids. The daily dose of the FS (2 tablets) provides 400 mg *Z. mays* extract, 600 mg *G. sylvestre* extract, 15 mg zinc, and 40 mg chromium. The ingredients used in this study are permitted for use as food supplement ingredients according to European legislation [30] and, as such, they are considered safe for human health and no toxicological assessment is requested to market the FS containing these ingredients. The placebo consisted of microcrystalline cellulose. To maintain blinding throughout the study, the placebo was designed to match the FS in its appearance, taste, and packaging. The tablets’ net weight was managed using Metrostat statistical software, following the Italian 25 October 1978 n. 690 Law and the standard UNI ISO 2859 guidelines. Upon receipt at the trial center, the shipments were registered and packing slip details were verified for accuracy, including batch numbers, manufacturing date, expiry date, manufacturer’s name, quantity, and storage conditions. Both the FS and placebo were stored in a locked cabinet within a locked room at room temperature. Entry and exit logbooks, as well as a FS accountability logbook, were maintained and periodically reviewed by the monitor. Dosage and administration were provided in accordance with the clinical study design outlined in the subsequent paragraph.

### 2.2. Clinical Trial Design and Ethical Approval

The study design included 81 participants that were randomly assigned into three groups (27 subjects for each group) by simple randomization (allocation ratio 1:1:1). The recruited subjects ingested two FS tablets, or one FS tablet and one placebo tablet, or two placebo tablets per day for the duration of the 90-day study period. The tablets were provided by the National Inter-University Consortium of Research in Innovative Pharmaceutical Technologies—TEFARCO—at no cost.

A double-blind, parallel-group, randomized, placebo-controlled trial was carried out to assess the benefits of the FS in lowering one or more risk factors for diabetes mellitus. Both the recruited subjects and the investigating physician were double blinded in this study. The participants were informed about the study both orally and in writing prior to submitting their written consent. The clinical trial was registered on the ISRCTN website (https://doi.org/10.1186/ISRCTN15697400, accessed on 31 May 2024). The study was carried out according to the 1964 Helsinki Declaration (as revised in 2000) and authorized by the Campania North Ethics Committee (protocol n. 1516, 14 April 2021). The protocol and the letter of intent were both approved by the Ethics Committee.

At the baseline (t0) and after 3 months (t1), the recruited subjects were submitted to blood sampling to evaluate the following biochemical parameters: fasting blood glucose (FBG); glycosylated hemoglobin (HbA1c); insulin (INS); HOMA-IR (homeostatic model assessment for insulin resistance); body mass index (BMI); total cholesterol (TC); low-density lipoprotein cholesterol (LDL-C); high-density lipoprotein cholesterol (HDL-C); plasma triglycerides (TG); aspartate transaminase (AST); alanine transaminase (ALT), and CRE. After enrollment of subjects, the total duration of the study was 90 days.

### 2.3. Participants and Recruiting Modalities

Participants in the study were recruited based on specific inclusion and exclusion criteria. A total of 81 subjects of either sex, aged 18–75 years, were enrolled and divided into three groups. Inclusion criteria required that participants exhibit impaired FBG ranging from 98 to 125 mg/dL. Subjects meeting criteria for high risk of cardiovascular events according to the parameters (sex, age, diabetes mellitus, smoking habit, systolic blood pressure, total cholesterol, HDL-cholesterol, and anti-hypertensive treatment) identified in the Progetto Cuore of the Istituto Superiore di Sanità, (http://www.cuore.iss.it/sopra/calc-rischio.asp, accessed on 31 May 2024) were excluded from the study. Individuals under current anti-diabetic treatment, and those using food supplements to decrease blood sugar in the two weeks prior to recruitment, were excluded from the study, as well as pregnant, suspected pregnant, or breastfeeding women; individuals who had donated blood within three months prior to recruitment; those who were not self-sufficient; unwilling to cooperate; those who had trouble in adhering to visit schedules; or were deemed unsuitable by the investigator due to the presence of other diseases.

### 2.4. Outcome of the Study

The primary outcome of the study was to evaluate the FS’s ability to reduce plasma glucose levels and maintain normal glucose metabolism in participants with mild impaired fasting glycemia. Examining the positive effects of the FS on lipid metabolism (TC, LDL-C, HDL-C, and TG) and body weight by evaluating body mass index (BMI) values at baseline and at the end of the experiment was the secondary outcome. At time t0 and time t1, ALT, AST, and CRE were also assessed in order to exclude renal and liver damage.

### 2.5. Tolerability

The participants were closely observed for the occurrence of any unfavorable side effects during the treatment. Individuals who were allergic to any of the ingredients in the FS were excluded from the trial.

### 2.6. Statistical Analysis

Sample size calculation was performed using three values of statistical power (1-β)—0.80, 0.95, and 0.99, a significance threshold level (α) of 0.05, and three effect sizes (Cohen’s f = 0.10, 0.30, and 0.45, respectively). Based on these parameters, the sample size was determined to be 81 participants, with 27 participants in each group. A mixed analysis of variance model with random intercept (random intercept LMM), in which the dependent variables represent the values of biochemical parameters, was deemed the most appropriate statistical analysis for this research. Due to individual variability in response to treatments with placebo (group 3) and FS treatment at two dosages (groups 1 and 2), the patient’s identification was entered as a random factor. The measurement, the therapy, and their interaction comprised the fixed factors.

## 3. Results

**Figure 1:**
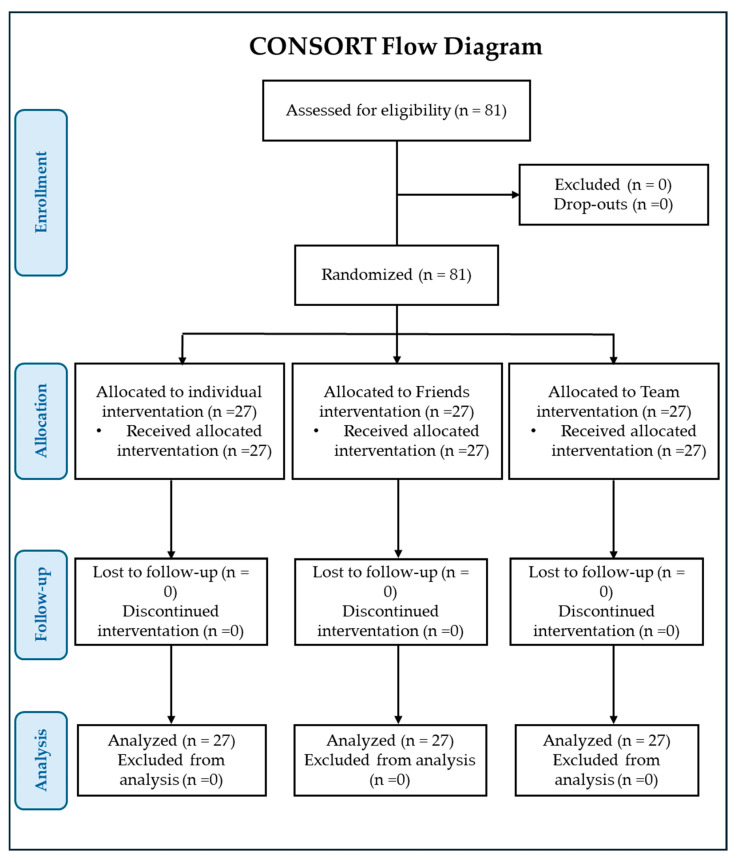
Consort PRO flow diagram.

The study flow chart, prepared following the CONSORT PRO reporting guidelines [31], is presented in Figure 1. The baseline characteristics of the subjects are summarized in Table 1. Of the 38 males enrolled in the study, 13 were allocated to group 1 and 13 were allocated to group 2; of the 43 females, 14 were allocated to group 1 and 14 were allocated to group 2. All received their daily treatment for 3 months. Group 1 received two tablets of the FS, group 2, one tablet of the FS and one tablet of placebo, and group 3 ingested two tablets of the placebo. The participants had similar sociodemographic characteristics and FBG levels (98–125 mg/dL).

Table 2 displays the descriptive statistics of the primary and secondary outcomes for each of the three experimental groups at the two measurement points (t0: baseline and t1: the third month of treatment).

**Figure 2:**
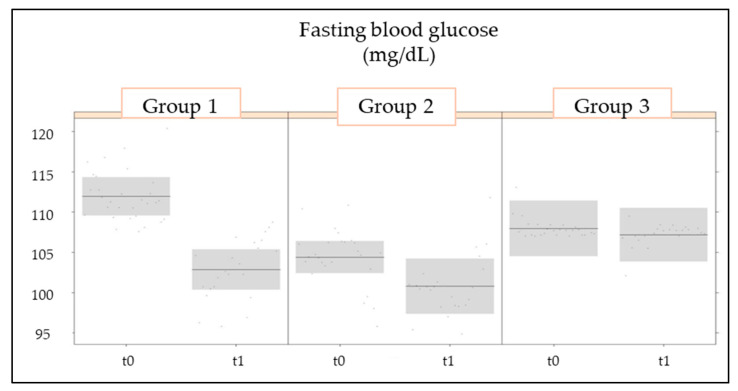
Changes in fasting blood sugar as a primary outcome in the treated experimental groups (groups 1 and 2) and placebo group (group 3) at the two evaluation timepoints (t0: baseline; t1: 3 months of treatment).

**Figure 3:**
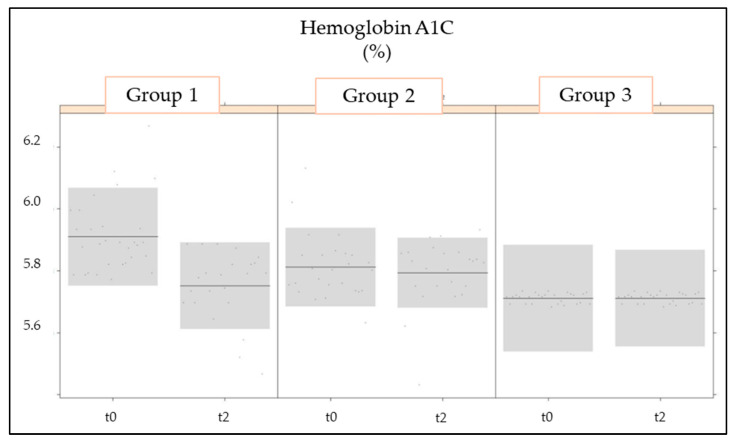
Changes in HbA1c as a primary outcome in the treated experimental groups (groups 1 and 2) and placebo group (group 3) at the two evaluation timepoints (t0: baseline; t1: 3 months of treatment).

The LMM model (Table 3) identified highly significant effects (*p* < 0.001) for the measurement values of FBG and for the interaction between group and measurement of FBG before (t0) and after (t1) the 3-month treatment. In fact, at t1, when compared to t0, blood glucose dropped significantly in groups 1 and 2 (Figure 2) and remained constant in group 3. A significant effect (*p* < 0.01) was also identified for the measurement and the group-measurement interaction of HbA1c (Figure 3). HbA1c values were significantly decreased in group 1 between t0 and t1 but not in the other two groups.

The analysis also revealed a significant effect in measurement values and group values of BMI, but not for the interaction between group and measurement (Table 3). This indicates that BMI decreased significantly between t0 and t1 and that the trend was the same in all groups, regardless of treatment with the FS (at both doses) or the placebo. While having a normal weight, the subjects recruited in group 3 had significantly lower BMI than the other groups. Table 2 displays descriptive statistics for the primary and secondary outcomes for each of the three experimental groups at the two measurement points (t0: baseline and t1: after the 3-month treatment). TC and HDL-C values followed the BMI trend, i.e., the model identified a significant effect between measurements and groups, but not in the interaction between group and measurement for both parameters, with a statistically significant decrease in TC levels and an increase in HDL-C cholesterol levels between t0 and t1. The trend was the same in all experimental groups (Table 2 and Table 3, respectively). Despite a slight decrease in HOMA-IR values observed after treatment in both group 1 and in group 2, for the other biochemical parameters (INS, HOMA-IR, TG, and LDL-C), no statistically significant difference was found in the data obtained before (t0) and after (t1) the treatments (Table 2 and Table 3). No statistically significant differences were identified before (t0) and after treatments (t1) with the FS or placebo (Table 2 and Table 3) in the parameters relative to liver (AST and ALT) and renal (CRE) function.

## 4. Discussion

Daily ingestion of the FS over a 3-month period in a randomized, placebo-controlled, double-blind clinical trial of subjects with mildly impaired glucose metabolism (98–125 mg/dL) was associated with a highly significant drop (*p* < 0.001) in FBG and HbA1c levels and good tolerability. Both at low and high doses, the effects of the FS on the other parameters examined were comparable to the effect of the placebo. In line with the inclusion criteria of the clinical trial protocol, the average values of FBG and HbA1c of the recruited subjects were lower than 126 mg/dL and 6%, respectively, corresponding to normal–high values of these parameters for healthy non-diabetic subjects. Although the decrease in HbA1c is small and was only recorded in the group treated with the highest dose of the FS, this result, together with the decrease in FBG, argues for this FS being a valid contender for the control of blood sugar in subjects with slight hyperglycemia. Hyperglycemia is a well-known risk factor of T2DM and induces inflammation (i.e., increase of proinflammatory cytokine release, proinflammatory signaling pathways, and release of the characteristic proinflammatory microparticles in monocytes/macrophages) [32], which, in turn, is linked to the development of diabetes complications such as macro- and microvascular diseases.

As diabetes mellitus is a multifactorial disease, primary prevention includes the ability to increase insulin secretion and cell glucose uptake, to inhibit glucose production and absorption through the inhibition of α-glucosidase and α-amylase enzymatic activities, to restore abnormal insulin pathway transmission, and to improve energy metabolism and inflammation [33]. Therefore, a FS consisting of several bioactive ingredients (i.e., plant extracts and micronutrients) with different mechanisms of action can be a useful strategy for maintaining glucose homeostasis by regulating numerous glucose metabolism pathways.

A large section of the results reported in the present study only agree in partially with those reported in the literature. However, it is difficult to compare the present results with the literature since, rather than the combined effects of extracts of *Z. mays* and *G. sylvestre* with zinc and chromium, the studies published to date have evaluated the effects of each component in isolation or in combinations containing only one of the components of the FS in the present study. In the following paragraphs, we discuss the data available concerning each of the components of the present FS.

The *Z. mays* extract from purple corn cobs is not clinically proven to be effective, but a preclinical study showed the extract (Moradyn^®^) can inhibit α-glucosidase in an in vitro system and may be involved in inhibiting the enzyme’s ability to digest carbohydrates in the intestinal tract. Furthermore, the phytocomplex of anthocyanins and polyphenols in Moradyn^®^ could prevent AGE formation, acting with potent antiglycative agents in in vitro model systems [17].

For its activity on glucose and lipid metabolism, *G. sylvestre* is the most widely studied component of this FS, both in preclinical and clinical studies. *G. sylvestre* may reduce glucose absorption in the small intestine through competition with gymnemic acid for the glucose receptors [34]. In addition, by inhibiting α-glucosidase, *G. sylvestre* extracts decrease carbohydrate digestion and stimulate insulin secretion without decreasing pancreatic β-cell viability in vitro [35,36]. Clinical studies [37,38,39] showed a significant impact of *G. sylvestre* on hyperglycemia indicators. Baskaran et al. [37] supplemented 22 T2DM patients with 400 mg/day *G. sylvestre* leaf extracts for 18–20 months. As a result, their levels of glycosylated plasma proteins, HbA1c, and blood glucose were significantly reduced. Likewise, diabetic patients (*n* = 58) supplemented with a *G. sylvestre* extract (500 mg/day) for three months experienced improvements in polyphagia, tiredness, fasting, and postprandial blood glucose levels and HbA1c [38]. Similarly, compared to 15 individuals with impaired glucose tolerance treated with a placebo, significant improvements in oral glucose tolerance tests, body weight, HbA1c, and LDL cholesterol levels were documented in 15 subjects with this metabolic abnormality treated with a *G. sylvestre* extract (600 mg/day) [39]. Finally, Zuñiga et al. [11] supplemented 24 adult MetS patients with a *G. sylvestre* extract (600 mg/day) for 12 weeks, with significant reductions in body weight, BMI, and VLDL levels and no changes in insulin secretion and sensitivity or blood glucose levels observed. In contrast with the subjects recruited in our study, those in the study reported above had already developed MetS. This may explain the discrepancy of our results with those reported by Zuñiga et al.

Recent findings from a systemic review and meta-analysis on the benefits of zinc supplementation in patients with diabetes mellitus indicated that zinc supplementation could be an effective strategy in the management of diabetes-associated hyperglycemia [40]. It may also reduce complications caused by diabetes-induced oxidative stress. A randomized controlled trial indicated beneficial effects of magnesium and zinc supplementation on a number of parameters and biomarkers related to the onset of type 2 diabetes and coronary heart disease, including FBG, HDL-C, and insulin levels [41]. These positive effects of zinc supplementation in diabetics are consistent with the knowledge that these patients may excrete significant amounts of zinc, and that increased oxidative stress and zinc depletion have a major detrimental influence on the development of diabetic problems [42].

There are conflicting conclusions on the direct association between chromium levels and the risk of MetS [43]. Studies in animals and in patients with T2DM and chromium deficiency support the concept that chromium is an essential micronutrient in insulin metabolism [44]. In addition to a reduction in lean body mass, chromium insufficiency is linked to increased blood levels of insulin, blood glucose, cholesterol, and TG. On the other hand, chromium supplementation improves insulin sensitivity, regulates blood sugar levels, and reduces cholesterol and triglyceride levels. The possibility should be explored that the benefits of chromium may vary depending on the chemical form of chromium used as a supplement and the individual characteristics of the subjects.

The protective effect of the FS under study in improving a normal glucose metabolism can be ascribed to the numerous plant bioactive compounds in the *Z. mays* extract. Its phytocomplex composition is reported by Ferron et al. [17] and includes anthocyanins (pelargonidin-3-*O*-glucoside, peonidin-3-*O*-glucoside, and cyanidin-3-*O*-glucoside), flavonoids (kaempferol, isorhamnetin derivatives, and myricetin), and hydroxycinnamic acid derivatives (5-O-feruloylquinic acid and a feruloyl derivative). The key mechanisms of the multifaceted effects of purple corn need to be analyzed thoroughly to elucidate its role in the efficacy of the FS analyzed here. The anti-diabetic activities of purple corn on the mechanisms that predispose to complications of diabetes mellitus have been thoroughly investigated. Purple corn has shown α-glucosidase-inhibitory activity [45]. Additionally, the Moradyn^®^ phytocomplex and its pure anthocyanin fraction have been shown to have antiglycative qualities and to efficiently prevent the synthesis of fructosamine [46]. Anthocyanins from purple maize pericarp reduced insulin resistance in 3T3-L1 adipocytes by increasing GLUT4 translocation and stimulating insulin signaling [47]. Moreover, the influence of an anthocyanin-rich purple maize pericarp extract on insulin secretion and hepatic glucose uptake in pancreatic cells and hepatocytes has also been investigated [48]. The extract may be able to improve type 2 diabetic comorbidities due to increased activity of glucokinase and free fatty acid receptor-1. In HIT-T15 (pancreatic beta cells), purple corn anthocyanins effectively prevented cell death [49]. Moreover, purple corn extract inhibited macrophage infiltration and accumulation in diabetic kidneys by controlling the mesangial IL-8-Tyk-STAT signaling pathway in human endothelial cells and THP-1 monocytes [50]. Purple corn rich in anthocyanins inhibited the growth of mesangial cells stimulated by high glucose levels and the deposition of matrix by controlling the TGF-β–SMAD and NF-κB pathways.

This knowledge is reinforced by the in vivo biological activity of purple corn extracts. In db/db mice, purple maize anthocyanins showed effective anti-hyperglycemic activity, while also lowering blood glucose and HbA1c levels [51]. Supplementation of rats with anthocyanin-rich purple maize resulted in reduced blood glucose levels, increased scores of HOMA-β (homeostasis model assessment of β-cell function) and HOMA-IS (homeostatic model assessment for insulin sensitivity), enhanced plasma glucagon-like peptide 1 (GLP1) and pancreatic glucagon-like peptide 1 receptor (GLP1R) levels, and improved pancreatic morphology [52]. Purple corn extract demonstrated anti-diabetic effects in C57BL/KsJ db/db mice through liver activation of AMP-activated protein kinase (AMPK), increased insulin production, and protection of pancreatic β-cells [53]. A study showed restoration of TNF-mRNA levels and attenuation of insulin resistance in mice when supplemented with a purple corn diet [54]. In db/db mice, an anthocyanin-rich purple maize extract alleviated severe albuminuria and lowered plasma glucose levels. Furthermore, by retarding the TGF-β signaling pathway, purple corn extract reduced the amount of collagen fiber accumulated in kidney glomeruli and the production of CTGF [55,56]. Purple waxy corn increased glutathione peroxidase activity and reduced lens opacity in diabetic experimental cataracts [57]. In streptozotocin-induced diabetic mice, the combination of purple waxy maize and ginger demonstrated a protective effect against diabetic eye problems, with an effective reduction of lens opacity [58]. A purple corn extract inhibited the production of VEGF and HIF-1a, hence preventing the glomerular angiogenesis of diabetic kidneys [59].

As a chronic inflammatory condition, obesity is a significant risk factor for the development of a number of chronic disorders, and adipose tissue plays a central role in the development of obesity [32]. An anthocyanin complex from purple waxy corn cobs, blue butterfly pea petals, and turmeric extract reduced the expression of oxidant-related genes (NF-κB and iNOS) and increased the expression of antioxidant-related genes (CAT, SOD, and GPx), exhibiting a protective effect against inflammation and periductal fibrosis in hamsters infected with *Opisthorchis viverini* [60]. By inhibiting the NF-κb and MAPK signaling pathways, purple maize anthocyanins and their metabolite proto-catechuic acid showed anti-inflammatory effects on AGES-induced human articular chondrocytes [61]. In a diet-induced obesity paradigm in mice, Tomay et al. [62] demonstrated the anti-obesity potential of purple corn cob extract. By increasing faecal butyric acid levels and hepatic SOD and GPx activity, with a decrease in lipid peroxidation and suppressed expression of TNFα, IL-6, iNOS, and NF-κB levels, purple maize anthocyanin efficiently demonstrated anti-obesity efficacy in C57BL/6 mice fed a high-fat diet [63]. Purple corn extract increased Akt phosphorylation and decreased macrophage infiltration into epididymal adipose tissue to mitigate the effects of high-fat diet-induced obesity and glucose intolerance [64]. Furthermore, anthocyanins from purple maize also activated the hepatic AMPK pathway, which resulted in reduced fatty acid synthase and increased fatty acid oxidation [65]. Supplementation of phenolic-rich water extract from purple maize pericarp for 12 weeks in a murine model attenuated obesity via modulation of toll-like receptor (TLR) and AMPK signaling pathways [47]. In white adipose tissue, purple corn lowers the mRNA levels of the sterol regulatory element binding protein-1 and downregulates the mRNA levels of enzymes involved in the production of fatty acids and triacylglycerol [54].

As far as *G. sylvestre* components are concerned, the bioactive constituents are represented by gymnemic acid, stigmasterol, quercetol, betaine, choline, and tri-methylamine. Specifically, gymnemic acid, a saponin, has been utilized for hundreds of years as a herbal treatment due to its hypoglycemic effects. It lowers blood glucose concentrations in diabetic patients, stimulates insulin production and its signal transduction, lessens endoplasmic reticulum stress [21,66,67,68,69], and attenuates inflammation and insulin resistance via PPAR-δ and NFκB-mediated signaling pathways [70]. Moreover, deacyl gymnemic acid treatment dramatically reduces insulin resistance and systolic blood pressure and enhances glycemic and lipid profiles as well as insulin sensitivity in a murine model of MetS [23].

The current study has its strengths and limitations. The efficacy and tolerability of the FS clearly emerges from the clinical trial. Over a prolonged time, dietary intervention may fail to control hyperglycemia. Having a FS with proven efficacy (in a randomized clinical trial) intended for subjects that are not candidates for conventional hypoglycemic drug therapy and that can act at with more than one mechanism of action due to its ingredient composition represents an option for physicians to help treat subjects at high risk of T2DM. The main limitation of the current study is that there was no follow-up period after the treatment ended, making it hard to assess if the glucose homeostasis had improved by the time the supplementation period ended. Moreover, lowering blood sugar levels does not necessarily reflect in hard endpoints (complications and mortality). More research is required to confirm the long-term effects of FSs based on *Z. mays* and *G. sylvestre* extracts, zinc, and chromium in improving hyperglycemia.

## 5. Conclusions

The results of this study demonstrate that a food supplement containing zinc, chromium, and extracts from *Z. mays* and *G. sylvestre*, when given for three months to subjects with mildly impaired fasting blood glucose concentrations, regulates glucose homeostasis by enhancing glucose metabolism. Larger-scale research is needed to support, in the long-term, the results of the current study and provide further knowledge of the use of this food supplement.

## Figures and Tables

**Table 1:** Characteristics of subjects enrolled in the study. Values presented as mean ± SD.

Characteristics of Enrolled Subjects	Group 1 (*n* = 27)	Group 2 (*n* = 27)	Group 3 (*n* = 27)
**Ethnicity** Caucasian	27	27	27
**Gender** Male (n) Female (n)	13 14	13 14	12 15
**Age (years)** Male Female	50.5 ± 11.1 54.3 ± 11.5	51.6 ± 13.2 53.8 ± 15.6	52.8 ± 14.0 57.5 ± 10.8
Mean age (years, range)	52.5 ± 13.4 (37–69)	52.5 ± 15.1 (34–68)	53.5 ± 14.1 (35–68)
Fasting glucose (mg/dL) (Range)	111.9 ± 16.5 (98–125)	104.4 ± 8.9 (98–121)	108.9 ± 8.0 (98–122)

**Group 1:** FS 2 tablets, placebo 0 tablets; **Group 2:** FS 1 tablet, placebo 1 tablet; **Group 3:** FS 0 tablets, placebo 2 tablets.

**Table 2:** Values (mean ± SD, minimum-maximum) of the primary and secondary outcomes (12 outcomes) evaluated at the two evaluation timepoints (t0: baseline and t1: at 3 months treatment) in the treatment groups and in the placebo group.

Variable	Group 1		Group 2		Group 3	
	t0	t1	t0	t1	t0	t1
FBG (mg/dL)	111.9 ± 16.5 (98–125)	100.2 ± 12.5 (78–125)	104.4 ± 8.9 (89–121)	100 ± 8.8 (92–125)	108 ± 8 (98–122)	107.2 ± 8.2 (96–124)
HbA1c (%)	5.9 ± 0.6 (5–8)	5.7 ± 0.4 (5–6.2)	5.8 ± 0.3 (5.2–6.4)	5.8 ± 0.3 (5–6.3)	5.7 ± 0.3 (5.1–6.2)	5.7 ± 0.3 (5.1–6.2)
INS (pmol/L)	13.2 ± 7.6 (2.3–42)	12.6 ± 5.7 (2.3–21.4)	9.7 ± 5.7 (2.6–22.5)	8.8 ± 5 (3.2–22.9)	10.4 ± 5.6 (3.5–21.5)	10 ± 5.3 (3.5–22)
HOMA-IR	3.6 ± 1.9 (0.6–10.9)	3.2 ± 1.4 (0.6–5.4)	2.6 ± 1.7 (0.8–6.4)	2.2 ± 1.4 (0.8–6)	2.8 ± 1.6 (0.8–6.5)	2.7 ± 1.5 (0.8–6.7)
BMI (kg/m^2^)	24.8 ± 4.6 (18–35.6)	23.7 ± 4 (18–32.3)	24.1 ± 3.7 (18–33.3)	23.6 ± 3.2 (18–30)	21.8 ± 2.9 (17–26)	21.7 ± 2.8 (17–26)
TC (mg/dL)	208.1 ± 33.1 (125–261)	200.2 ± 28.1 (122–254)	218.8 ± 37.6 (130–280)	206.4 ± 33 (125–260)	223.9 ± 21.5 (178–256)	223.4 ± 21.4 (190–260)
LDL-C (mg/dL)	105.3 ± 22 (60–172)	99.1 ± 21.2 (79–164)	105.6 ± 27.9 (73–221)	96.2 ± 12.2 (63–123)	105.3 ± 16.1 (79–145)	107.7 ± 18.9 (83–151)
HDL-C (mg/dL)	45.8 ± 10.4 (23–65)	48.3 ± 12.6 (24–70)	54.4 ± 13.4 (34–83)	56.2 ± 15.1 (35–87)	50 ± 8.9 (35–67)	50.7 ± 9.5 (34–69)
TG (mg/dL)	162.6 ± 48.6 (4.8–257)	180.4 ± 61.3 (67–349)	146.2 ± 45 (35–190)	141 ± 45.2 (40–185)	170.4 ± 14 (143–190)	167.8 ± 15.9 (139–200)
AST (mg/dL)	22.9 ± 8.3 (5–40)	23 ± 7.5 (13–38)	22.6 ± 5.3 (16–33)	23.7 ± 4.7 (16–32)	25.6 ± 4.3 (18–37)	26.6 ± 4.9 (18–40)
ALT (U/L)	27.2 ± 8.2 (12–41)	28.6 ± 7.5 (11–41)	27.7 ± 8.8 (11–44)	30 ± 10.4 (12–45)	32.3 ± 5.1 (21–41)	32.1 ± 4.9 (22–41)
CRE (mg/dL)	1 ± 0.2 (0.7–1.3)	5.4 ± 20.9 (0.8–101.3)	1 ± 0.2 (0.6–1.3)	1 ± 0.1 (0.7–1.2)	1.1 ± 0.1 (0.9–1.2)	1.1 ± 0.1 (0.9–1.3)

**Group 1:** 2 tablets of the FS, no placebo tablet; **Group 2:** 1 tablet of the FS, 1 tablet of placebo; **Group 3:** 0 FS tablet, 2 tablets of placebo. FBG (fasting blood glucose); HbA1c (glycosylated hemoglobin); INS (insulin); HOMA-IR (homeostatic model assessment for insulin resistance); BMI (body mass index); TC (total cholesterol); LDL-C (low-density lipoprotein cholesterol); HDL-C (high density lipoprotein cholesterol); TG (plasma triglycerides); AST (aspartate transaminase); ALT (alanine transaminase); CRE (creatinine).

**Table 3:** LMM model results for variables associated with the primary and secondary outcomes.

Variable	Model	Num	Den	F	*p*
FBG	Measurement	1	68	44.367	**<0.001**
	Group	2	76	1.507	0.228
	Group for measure	2	68	13.089	**<0.001**
HbA1c	Measurement	1	70	7.850	**0.007**
	Group	2	78	0.621	0.540
	Group for measure	2	70	5.565	**0.006**
INS	Measurement	1	67	0.635	0.428
	Group	2	73	3.641	**0.031**
	Group for measure	2	67	0.117	0.890
HOMA-IR	Measurement	1	68	2.162	0.146
	Group	2	73	3.586	0.033
	Group for measure	2	68	0.281	0.756
BMI	Measurement	1	71	4.811	**0.032**
	Group	2	80	4.839	**0.010**
	Group for measure	2	71	1.632	0.203
TC	Measurement	1	71	9.111	**0.004**
	Group	2	77	4.412	**0.015**
	Group for measure	2	71	2.149	0.124
LDL-C	Measurement	1	70	3.069	0.084
	Group	2	76	0.654	0.523
	Group for measure	2	70	2.421	0.096
HDL-C	Measurement	1	72	5.778	**0.019**
	Group	2	81	3.564	**0.033**
	Group for measure	2	72	0.672	0.514
TG	Measurement	1	73	0.188	0.666
	Group	2	80	3.619	**0.031**
	Group for measure	2	73	2.105	0.129
AST	Measurement	1	72	0.469	0.496
	Group	2	80	3.336	**0.041**
	Group for measure	2	72	1.892	0.158
ALT	Measurement	1	73	0.747	0.390
	Group	2	79	3.692	**0.029**
	Group for measure	2	73	1.031	0.362
CRE	Measurement	1	82	1.324	0.253
	Group	2	82	1.295	0.279
	Group for measure	2	82	1.313	0.275

FBG (fasting blood glucose); HbA1c (glycosylated hemoglobin); INS (insulin); HOMA-IR (homeostatic model assessment for insulin resistance); BMI (body mass index); TC (total cholesterol); LDL-C (low-density lipoprotein cholesterol); HDL-C (high density lipoprotein cholesterol); TG (plasma triglycerides); AST (aspartate transaminase); ALT (alanine transaminase); CRE (creatinine).

## Data Availability

The original contributions presented in the study are included in the article, and further inquiries can be directed to the corresponding authors.

## References

[1] De Filippis A., Ullah H., Baldi A., Dacrema M., Esposito C., Garzarella E.U., Santarcangelo C., Tantipongpiradet A., Daglia M. (2020). Gastrointestinal disorders and metabolic syndrome: Dysbiosis as a key link and common bioactive dietary components useful for their treatment. Int. J. Mol. Sci..

[2] Ullah H., De Filippis A., Khan H., Xiao J., Daglia M. (2020). An overview of the health benefits of Prunus species with special reference to metabolic syndrome risk factors. Food Chem. Toxicol..

[3] Ambroselli D., Masciulli F., Romano E., Catanzaro G., Besharat Z.M., Massari M.C., Ferretti E., Migliaccio S., Izzo L., Ritieni A. (2023). New advances in metabolic syndrome, from prevention to treatment: The role of diet and food. Nutrients.

[4] Ullah H., Sommella E., Santarcangelo C., D’Avino D., Rossi A., Dacrema M., Minno A.D., Di Matteo G., Mannina L., Campiglia P. (2022). Hydroethanolic extract of Prunus domestica L.: Metabolite profiling and in vitro modulation of molecular mechanisms associated to cardiometabolic diseases. Nutrients.

[5] Rochlani Y., Pothineni N.V., Kovelamudi S., Mehta J.L. (2017). Metabolic syndrome: Pathophysiology, management, and modulation by natural compounds. Ther. Adv. Cardiovasc. Dis..

[6] Castro-Barquero S., Ruiz-Leon A.M., Sierra-Perez M., Estruch R., Casas R. (2020). Dietary strategies for metabolic syndrome: A comprehensive review. Nutrients.

[7] Harrison S., Couture P., Lamarche B. (2020). Diet quality, saturated fat and metabolic syndrome. Nutrients.

[8] Myers J., Kokkinos P., Nyelin E. (2019). Physical activity, cardiorespiratory fitness, and the metabolic syndrome. Nutrients.

[9] Rask Larsen J., Dima L., Correll C.U., Manu P. (2018). The pharmacological management of metabolic syndrome. Expert. Rev. Clin. Pharmacol..

[10] Ben Salem M., Affes H., Ksouda K., Dhouibi R., Sahnoun Z., Hammami S., Zeghal K.M. (2015). Pharmacological studies of Artichoke leaf extract and their health benefits. Plant Foods Hum. Nutr..

[11] Fomenko E.V., Chi Y. (2016). Mangiferin modulation of metabolism and metabolic syndrome. Biofactors.

[12] He L., Xing Y., Ren X., Zheng M., Yu S., Wang Y., Xiu Z., Dong Y. (2022). Mulberry leaf extract improves metabolic syndrome by alleviating lipid accumulation in vitro and in vivo. Molecules.

[13] Roshan H., Nikpayam O., Sedaghat M., Sohrab G. (2018). Effects of green coffee extract supplementation on anthropometric indices, glycaemic control, blood pressure, lipid profile, insulin resistance and appetite in patients with the metabolic syndrome: A randomised clinical trial. Br. J. Nutr..

[14] Zuniga L.Y., Gonzalez-Ortiz M., Martinez-Abundis E. (2017). Effect of *Gymnema sylvestre* administration on metabolic syndrome, insulin sensitivity, and insulin secretion. J. Med. Food.

[15] Panchal S.K., Wanyonyi S., Brown L. (2017). Selenium, vanadium, and chromium as micronutrients to improve metabolic syndrome. Curr. Hypertens. Rep..

[16] Shi Y., Zou Y., Shen Z., Xiong Y., Zhang W., Liu C., Chen S. (2020). Trace elements, PPARs, and metabolic syndrome. Int. J. Mol. Sci..

[17] Ferron L., Colombo R., Mannucci B., Papetti A. (2020). A New Italian purple corn variety (Moradyn) byproduct extract: Antiglycative and hypoglycemic in vitro activities and preliminary bioaccessibility studies. Molecules.

[18] Amin E., Abdel-Bakky M.S., Darwish M.A., Mohammed H.A., Chigurupati S., Qureshi K.A., Hassan M.H.A. (2022). The glycemic control potential of some Amaranthaceae plants, with particular reference to in vivo antidiabetic potential of Agathophora alopecuroides. Molecules.

[19] Qiu Q., Zhen H.S., Wei Y.F., Zhen D.D. (2017). Research progress on effective substance and quality analysis of *Gymnema sylvestre*(Retz.) schult. Chin. J. Ethnomed. Ethnopharm..

[20] Devangan S., Varghese B., Johny E., Gurram S., Adela R. (2021). The effect of *Gymnema sylvestre* supplementation on glycemic control in type 2 diabetes patients: A systematic review and meta-analysis. Phytother. Res..

[21] Pothuraju R., Sharma R.K., Chagalamarri J., Jangra S., Kumar Kavadi P. (2014). A systematic review of *Gymnema sylvestre* in obesity and diabetes management. J. Sci. Food Agric..

[22] Muzaffar H., Qamar I., Bashir M., Jabeen F., Irfan S., Anwar H. (2023). *Gymnema sylvestre* supplementation restores normoglycemia, corrects dyslipidemia, and transcriptionally modulates pancreatic and hepatic gene expression in alloxan-induced hyperglycemic rats. Metabolites.

[23] Bhansali S., Shafiq N., Pandhi P., Singh A.P., Singh I., Singh P.K., Sharma S., Malhotra S. (2013). Effect of a deacyl gymnemic acid on glucose homeostasis & metabolic parameters in a rat model of metabolic syndrome. Indian J. Med. Res..

[24] Tamura Y. (2021). The role of zinc homeostasis in the prevention of diabetes mellitus and cardiovascular diseases. J. Atheroscler. Thromb..

[25] Bjorklund G., Dadar M., Pivina L., Dosa M.D., Semenova Y., Aaseth J. (2020). The role of zinc and copper in insulin resistance and diabetes mellitus. Curr. Med. Chem..

[26] Cruz K.J.C., de Oliveira A.R.S., Morais J.B.S., Severo J.S., Mendes P.M.V., de Sousa Melo S.R., de Sousa G.S., Marreiro D.D.N. (2018). Zinc and insulin resistance: Biochemical and molecular aspects. Biol. Trace Elem. Res..

[27] Morais J.B.S., Severo J.S., Beserra J.B., de Oiveira A.R.S., Cruz K.J.C., de Sousa Melo S.R., do Nascimento G.V.R., de Macedo G.F.S., do Nascimento Marreiro D. (2019). Association between cortisol, insulin resistance and zinc in obesity: A mini-review. Biol. Trace Elem. Res..

[28] Jeejeebhoy K.N., Chu R.C., Marliss E.B., Greenberg G.R., Bruce-Robertson A. (1977). Chromium deficiency, glucose intolerance, and neuropathy reversed by chromium supplementation, in a patient receiving long-term total parenteral nutrition. Am. J. Clin. Nutr..

[29] Vincent J.B. (2019). Effects of chromium supplementation on body composition, human and animal health, and insulin and glucose metabolism. Curr. Opin. Clin. Nutr. Metab. Care.

[30] Directive E.U. (2002). Directive 2002/46/EC of the European Parliament and of the Council of 10 June 2002 on the approximation of the laws of the Member States relating to food supplements. Off. J. Eur. Communities Legis..

[31] Calvert M., Blazeby J., Altman D.G., Revicki D.A., Moher D., Brundage M.D., Group C.P. (2013). Reporting of patient-reported outcomes in randomized trials: The CONSORT PRO extension. JAMA.

[32] Myette-Côté É., Durrer C., Neudorf H., Bammert T.D., Botezelli J.D., Johnson J.D., DeSouza C.A., Little J.P. (2018). The effect of a short-term low-carbohydrate, high-fat diet with or without postmeal walks on glycemic control and inflammation in type 2 diabetes: A randomized trial. Am. J. Physiol. Regul. Integr. Comp. Physiol..

[33] Rosen E.D. (2016). Epigenomic and transcriptional control of insulin resistance. J. Intern. Med..

[34] Yeh G.Y., Eisenberg D.M., Kaptchuk T.J., Phillips R.S. (2003). Systematic review of herbs and dietary supplements for glycemic control in diabetes. Diabetes Care.

[35] El-Shafey A., El-Ezabi M., Selim M., Ouda H., Ibrahim D. (2013). Effect of *Gymnema sylvestre* R. Br. leaves extract on certain physiological parameters of diabetic rats. J. King Saud Univ. Sci..

[36] Liu B., Asare-Anane H., Al-Romaiyan A., Huang G., Amiel S.A., Jones P.M., Persaud S.J. (2009). Characterisation of the insulinotropic activity of an aqueous extract of *Gymnema sylvestre* in mouse beta-cells and human islets of Langerhans. Cell. Physiol. Biochem..

[37] Baskaran K., Kizar Ahamath B., Radha Shanmugasundaram K., Shanmugasundaram E.R. (1990). Antidiabetic effect of a leaf extract from *Gymnema sylvestre* in non-insulin-dependent diabetes mellitus patients. J. Ethnopharmacol..

[38] Kumar S.N., Mani U.V., Mani I. (2010). An open label study on the supplementation of *Gymnema sylvestre* in type 2 diabetics. J. Diet. Suppl..

[39] Gaytan Martinez L.A., Sanchez-Ruiz L.A., Zuniga L.Y., Gonzalez-Ortiz M., Martinez-Abundis E. (2021). Effect of *Gymnema sylvestre* administration on glycemic control, insulin secretion, and insulin sensitivity in patients with impaired glucose tolerance. J. Med. Food.

[40] Wang X., Wu W., Zheng W., Fang X., Chen L., Rink L., Min J., Wang F. (2019). Zinc supplementation improves glycemic control for diabetes prevention and management: A systematic review and meta-analysis of randomized controlled trials. Am. J. Clin. Nutr..

[41] Hamedifard Z., Farrokhian A., Reiner Z., Bahmani F., Asemi Z., Ghotbi M., Taghizadeh M. (2020). The effects of combined magnesium and zinc supplementation on metabolic status in patients with type 2 diabetes mellitus and coronary heart disease. Lipids Health Dis..

[42] Barman S., Srinivasan K. (2022). Diabetes and zinc dyshomeostasis: Can zinc supplementation mitigate diabetic complications?. Crit. Rev. Food Sci. Nutr..

[43] Bai J., Xun P., Morris S., Jacobs D.R., Liu K., He K. (2015). Chromium exposure and incidence of metabolic syndrome among American young adults over a 23-year follow-up: The CARDIA trace element study. Sci. Rep..

[44] Khodavirdipour A., Haddadi F., Keshavarzi S. (2020). Chromium supplementation; negotiation with diabetes mellitus, hyperlipidemia and depression. J. Diabetes Metab. Disord..

[45] Luna-Vital D., Luzardo-Ocampo I., Cuellar-Nunez M.L., Loarca-Pina G., Gonzalez de Mejia E. (2020). Maize extract rich in ferulic acid and anthocyanins prevents high-fat-induced obesity in mice by modulating SIRT1, AMPK and IL-6 associated metabolic and inflammatory pathways. J. Nutr. Biochem..

[46] Lee C.H., Garcia H.S., Parkin K.L. (2010). Bioactivities of kernel extracts of 18 strains of maize (*Zea mays*). J. Food Sci..

[47] Luna-Vital D., Weiss M., Gonzalez de Mejia E. (2017). Anthocyanins from purple corn ameliorated tumor necrosis factor-alpha-induced inflammation and insulin resistance in 3T3-L1 adipocytes via activation of insulin signaling and enhanced GLUT4 translocation. Mol. Nutr. Food Res..

[48] Luna-Vital D.A., Gonzalez de Mejia E. (2018). Anthocyanins from purple corn activate free fatty acid-receptor 1 and glucokinase enhancing in vitro insulin secretion and hepatic glucose uptake. PLoS ONE.

[49] Hong S.H., Heo J.I., Kim J.H., Kwon S.O., Yeo K.M., Bakowska-Barczak A.M., Kolodziejczyk P., Ryu O.H., Choi M.K., Kang Y.H. (2013). Antidiabetic and beta cell-protection activities of purple corn anthocyanins. Biomol. Ther..

[50] Kang M.K., Li J., Kim J.L., Gong J.H., Kwak S.N., Park J.H., Lee J.Y., Lim S.S., Kang Y.H. (2012). Purple corn anthocyanins inhibit diabetes-associated glomerular monocyte activation and macrophage infiltration. Am. J. Physiol. Renal Physiol..

[51] Colombo R., Ferron L., Papetti A. (2021). Colored corn: An up-date on metabolites extraction, health implication, and potential use. Molecules.

[52] Chayati I., Sunarti S., Marsono Y., Astuti M. (2019). Anthocyanin extract of purple corn improves hyperglycemia and insulin resistance of rats fed high fat and fructose diet via GLP1 and GLP1R mechanism. J. Food Nutr. Res..

[53] Huang B., Wang Z., Park J.H., Ryu O.H., Choi M.K., Lee J.Y., Kang Y.H., Lim S.S. (2015). Anti-diabetic effect of purple corn extract on C57BL/KsJ db/db mice. Nutr. Res. Pract..

[54] Tsuda T., Horio F., Uchida K., Aoki H., Osawa T. (2003). Dietary cyanidin 3-O-beta-D-glucoside-rich purple corn color prevents obesity and ameliorates hyperglycemia in mice. J. Nutr..

[55] Li J., Kang M.K., Kim J.K., Kim J.L., Kang S.W., Lim S.S., Kang Y.H. (2012). Purple corn anthocyanins retard diabetes-associated glomerulosclerosis in mesangial cells and db/db mice. Eur. J. Nutr..

[56] Li J., Lim S.S., Lee J.Y., Kim J.K., Kang S.W., Kim J.L., Kang Y.H. (2012). Purple corn anthocyanins dampened high-glucose-induced mesangial fibrosis and inflammation: Possible renoprotective role in diabetic nephropathy. J. Nutr. Biochem..

[57] Thiraphatthanavong P., Wattanathorn J., Muchimapura S., Wipawee T.M., Wannanon P., Terdthai T.U., Suriharn B., Lertrat K. (2014). Preventive effect of *Zea mays* L. (purple waxy corn) on experimental diabetic cataract. Biomed. Res. Int..

[58] Thiraphatthanavong P., Wattanathorn J., Muchimapura S., Thukham-mee W., Lertrat K., Suriharn B. (2014). The combined extract of purple waxy corn and ginger prevents cataractogenesis and retinopathy in streptozotocin-diabetic rats. Oxid. Med. Cell. Longev..

[59] Kang M.K., Lim S.S., Lee J.Y., Yeo K.M., Kang Y.H. (2013). Anthocyanin-rich purple corn extract inhibit diabetes-associated glomerular angiogenesis. PLoS ONE.

[60] Intuyod K., Priprem A., Limphirat W., Charoensuk L., Pinlaor P., Pairojkul C., Lertrat K., Pinlaor S. (2014). Anti-inflammatory and anti-periductal fibrosis effects of an anthocyanin complex in Opisthorchis viverrini-infected hamsters. Food Chem. Toxicol..

[61] Chuntakaruk H., Kongtawelert P., Pothacharoen P. (2021). Chondroprotective effects of purple corn anthocyanins on advanced glycation end products induction through suppression of NF-kappaB and MAPK signaling. Sci. Rep..

[62] Tomay F., Marinelli A., Leoni V., Caccia C., Matros A., Mock H.P., Tonelli C., Petroni K. (2019). Purple corn extract induces long-lasting reprogramming and M2 phenotypic switch of adipose tissue macrophages in obese mice. J. Transl. Med..

[63] Wu T., Guo X., Zhang M., Yang L., Liu R., Yin J. (2017). Anthocyanins in black rice, soybean and purple corn increase fecal butyric acid and prevent liver inflammation in high fat diet-induced obese mice. Food Funct..

[64] Hyunchae J., Chai-hee K., Yejoo L., Soon-kwon K., Myoung-Sool D. (2017). Anti-diabetic and anti-inflammatory effects of purple corn extract in high-fat diet induced obesity mice. Korean J. Food Nutr..

[65] Xu H., Liu M., Liu H., Zhao B., Zheng M., Liu J. (2021). Anthocyanins from purple corn ameliorated obesity in high fat diet-induced obese mice through activating hepatic AMPK. J. Funct. Foods.

[66] Izutani Y., Murai T., Imoto T., Ohnishi M., Oda M., Ishijima S. (2005). Gymnemic acids inhibit rabbit glyceraldehyde-3-phosphate dehydrogenase and induce a smearing of its electrophoretic band and dephosphorylation. FEBS Lett..

[67] Di Fabio G., Romanucci V., De Marco A., Zarrelli A. (2014). Triterpenoids from *Gymnema sylvestre* and their pharmacological activities. Molecules.

[68] Renga B., Festa C., De Marino S., Di Micco S., D’Auria M.V., Bifulco G., Fiorucci S., Zampella A. (2015). Molecular decodification of gymnemic acids from *Gymnema sylvestre*. Discovery of a new class of liver X receptor antagonists. Steroids.

[69] Li Y., Sun M., Liu Y., Liang J., Wang T., Zhang Z. (2019). Gymnemic acid alleviates type 2 diabetes mellitus and suppresses endoplasmic reticulum stress in vivo and in vitro. J. Agric. Food Chem..

[70] Li Y., Xiao Y., Gao W., Pan J., Zhao Q., Zhang Z. (2019). Gymnemic acid alleviates inflammation and insulin resistance via PPARδ- and NFκB-mediated pathways in db/db mice. Food Funct..

